# ﻿A natural hybrid of *Sindora* (Fabaceae, Detarioideae) from Singapore

**DOI:** 10.3897/phytokeys.190.79185

**Published:** 2022-02-23

**Authors:** Le Min Choo, Adrian Hock Beng Loo, Wee Foong Ang, Kenneth Boon Hwee Er

**Affiliations:** 1 National Parks Board, 1 Cluny Road, 259569, Singapore, Singapore National Parks Board Singapore Singapore

**Keywords:** Caesalpinioideae, Changi, ddRAD, new hybrid, *Sindoracoriacea*, *Sindoraechinocalyx*

## Abstract

Sindora×changiensis L.M.Choo, Loo, W.F.Ang & K.Er is a new hybrid from the subfamily Detarioideae in Fabaceae. This is the first reported instance of natural hybridisation in *Sindora*. Based on population genetics analyses using ddRAD and morphological observations, this taxon represents a fertile hybrid between *Sindoracoriacea* and *Sindoraechinocalyx*. This new hybrid is so far only known to occur naturally from Changi at the north-eastern coast of Singapore. It has pods that are sparsely spiny. This is intermediate between the smooth, non-spiny pods of *S.coriacea*, and the densely spiny pods of *S.echinocalyx*. The calyx is smooth and unarmed, resembling *S.coriacea*. Last but not least, the ovary is entirely pubescent, different from *S.coriacea* and *S.echinocalyx*. The ovary of *S.coriacea* has a glabrous patch in the middle, while that of *S.echinocalyx* has minute spines protruding from the dense pubescence. A taxonomic description and an updated key to the *Sindora* of Singapore and Peninsular Malaysia are also provided.

## ﻿Introduction

*Sindora* Miq. is a genus of the legume family (Fabaceae: Detarioideae) and consists of 20–22 species. It has a paleotropical distribution, with one species from West Central Africa, and the rest from Asia, which are distributed from Southern China to Southeast Asia, and to as far west as the Philippines (de Wit 1949; Chen et al. 2010; Choo and Ngo 2020). The genus can be easily recognised in the field by its characteristic pods, often with spines originating from modified glands, and dark-coloured seeds with a large yellow or reddish-brown aril of about the same size as the seed itself (de Wit 1949; Sosef 1993).

Previous work on *Sindora* in Singapore and Peninsular Malaysia reported five native species to the region, i.e. *Sindoracoriacea* (Baker) Prain, *Sindoraechinocalyx* Prain, *Sindorasiamensis* Teijsm. ex Miq., *Sindoravelutina* Baker, *Sindorawallichii* Benth. (Choo and Ngo 2020). Of these, four species, *Sindoracoriacea*, *S.echinocalyx*, *S.velutina* and *S.wallichii* are present in both Singapore and Peninsular Malaysia, while *S.siamensis* is found only in northern Peninsular Malaysia, Thailand, Cambodia, Laos and Vietnam, and is only present as a cultivated tree in Singapore. In Singapore, the four native species can be found in the remaining patches of primary and mature secondary rainforest. *S.coriacea* and *S.wallichii* are relatively more abundant than *S.velutina* and *S.echinocalyx*, which are comparatively rarer in the wild in Singapore. *S.velutina* is found only in the primary rainforest at Bukit Timah Nature Reserve, while *S.echinocalyx* may be more associated with hillsides and coastal areas (Choo and Ngo 2020). Recent field observations of mature *Sindora* trees in Singapore revealed a particular 27 m-tall mature tree from Changi at the north-eastern coast of the island, which could not be satisfactorily identified to species using the taxonomic key in Choo and Ngo (2020).

From a combination of population genetics and morphological evidence, we found that this particular tree represents a natural hybrid between *Sindoracoriacea* and *Sindoraechinocalyx*, and is the first recorded instance of hybridisation in *Sindora*. We describe this new hybrid as Sindora×changiensis L.M.Choo, Loo, W.F.Ang & K.Er, and further explain the characteristics that distinguish this new hybrid from its parent species.

## ﻿Materials and methods

### ﻿Population genetics analysis

Double-digest RAD-sequencing (ddRAD) was carried out to investigate the hybrid origin of Sindora×changiensis. Of the four native species of *Sindora* in Singapore, *S.echinocalyx*, one of the likely parents as an individual of *S.echinocalyx*, was formerly collected from Changi, Singapore in 1893 as a herbarium specimen, so this species was known to exist in the area. *S.coriacea* was selected as the next most likely parent because of its smooth and spineless pods, as the hybrid had sparsely spiny pods and other characteristics which were intermediate between, or a mix between, these two species (see Results for more details). The other two native species, *S.velutina* and *S.wallichii*, were unlikely to have been the other parent because they both have spiny pods, and would not have resulted in the sparsely spiny pods of the hybrid if they had hybridised with *S.echinocalyx*.

A total of 14 individuals from Singapore were sampled and sequenced, consisting of six *S.coriacea* individuals, four *S.echinocalyx* individuals and three S.×changiensis individuals (the mature tree and two other seedlings from the tree). Leaf material from each tree was silica-dried and the resulting herbarium specimens were deposited in SING. Details of the specimens used are available in Table 1.

**Table 1.** Details of 14 specimens sequenced in the population genetics analysis. (BTNR = Bukit Timah Nature Reserve, CCNR = Central Catchment Nature Reserve).

Sample	Species	Collector	Voucher	Locality	Notes
Sind002	*Sindoracoriacea*	Choo, L.M. & Ngo, K.M.	SING2019-840	BTNR	Wild
Sind063	*Sindoracoriacea*	Ng, X.Y.	SING2021-396	CCNR	Wild
Sind064	*Sindoracoriacea*	Niissalo, M.A. & Choo, L.M.	SING2021-599	CCNR	Wild
Sind065	*Sindoracoriacea*	Niissalo, M.A. & Choo, L.M.	SING2021-600	CCNR	Wild
Sind066	*Sindoracoriacea*	Niissalo, M.A. & Choo, L.M.	SING2021-601	CCNR	Wild
Sind067	*Sindoracoriacea*	Niissalo, M.A. & Choo, L.M.	SING2021-602	CCNR	Wild
Sind068	*Sindoracoriacea*	Niissalo, M.A. & Choo, L.M.	SING2021-603	CCNR	Wild
Sind006	Sindora×changiensis	Choo, L.M.	SING2020-649	Changi	Seedling of Sind019
Sind017	Sindora×changiensis	Choo, L.M.	SING2020-650	Changi	Seedling of Sind019
Sind019	Sindora×changiensis	Choo, L.M. et al.	SING2021-265	Changi	Wild, Mature tree
Sind010	*Sindoraechinocalyx*	Choo, L.M. et al.	SING2020-1212	Changi	Cultivated
Sind011	*Sindoraechinocalyx*	Choo, L.M. et al.	SING2020-1213	Changi	Cultivated
Sind020	*Sindoraechinocalyx*	Choo, L.M. et al.	SING2021-266	Changi	Cultivated
Sind021	*Sindoraechinocalyx*	Choo, L.M. et al.	SING2021-267	Changi	Cultivated

Genomic DNA extraction was done using the CTAB method (Doyle and Doyle 1987). ddRAD-sequencing libraries were prepared using the methods and barcodes first published by Peterson et al. (2012), and further modified by Niissalo et al. (2018, 2020). 600 ng of genomic DNA was used in the restriction digest by ApeKI and PstI, followed by a size selection using 5% SeraMag Magnetic Carboxylate-Modified Microparticles (Cytiva, USA). PCR was carried out using KAPA High-Fidelity DNA Polymerase (Roche, USA) and the concentration and library size of each sample was measured, before they were pooled together in equimolar volumes for sequencing.

The pooled libraries were sequenced by NovogeneAIT Genomics (Singapore) using Illumina NovaSeq. Sequences were demultiplexed using the process_radtags function in STACKS v1.37 (Catchen et al. 2013). STACKS was also used for de novo sequence assembly and single nucleotide polymorphism (SNP) discovery. Sequences with a minimum depth of 15 were retained, and one SNP per loci was used using the setting – write-random-snps, with SNPs present in at least 12 out of the 14 individuals selected for further analysis. Across the 14 individuals, a total of 19,856 SNPs were recovered.

To visualise the relationships between *Sindoracoriacea*, *S.echinocalyx* and S.×changiensis, a neighbour-net plot with uncorrected p-distances was made using the Neighbour-Net function in SPLITSTREE v4.17.1 (Huson and Bryant 2006). A STRUCTURE analysis (Pritchard et al. 2000) was carried out to find out the number of genetic clusters, or K-value, in the samples sequenced. We used 10,000 randomly selected independent SNPs from the 19,856 SNPs available, with a burn-in of 10,000. This was followed by 100,000 Markov Chain Monte Carlo (MCMC) repetitions, with the values of K from 1 to 5, which was 3 more than the expected number of two populations. A total of 30 STRUCTURE iterations were run, and the optimal value of K was determined by calculating the mean *L* (K) and the standard deviation of *L* (K) for each value of K, and the ΔK for K = 2 to K = 5 using STRUCTURE HARVESTER (Earl and von Holdt 2012).

### ﻿Morphological observations

Morphological observations of the hybrid Sindora×changiensis were made using both freshly collected and dried herbarium specimens in SING. The traits of *S.coriacea* and *S.echinocalyx* were obtained from the accounts of both species in their recent treatment in Choo and Ngo (2020), which was mainly based upon an extensive study of herbarium specimens from SING and KEP, and supplemented with measurements of recent specimens in SING collected between 2020 and 2021. Measurements of microscopic structures were made using a dissecting microscope with a calibrated eyepiece. Fresh flowers were used for the measurements where available, otherwise rehydrated flowers from dried herbarium specimens were used.

## ﻿Results

### ﻿Population genetics

**Figure 1.**
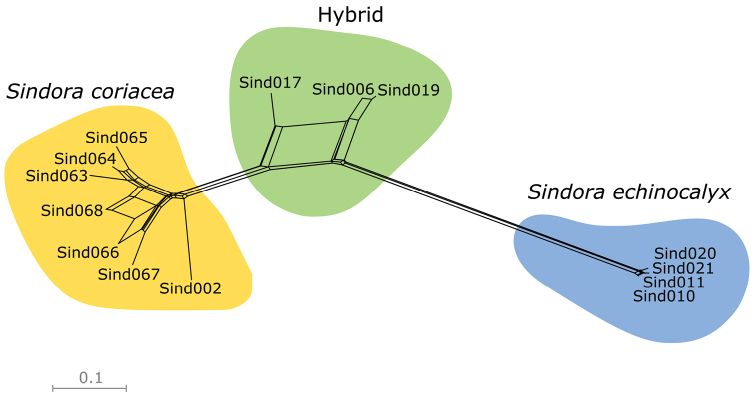
A neighbour-net plot with uncorrected p-distances, constructed in SPLITSTREE using the SNP dataset of all 14 individuals of *Sindora* sequenced.

**Figure 2.**
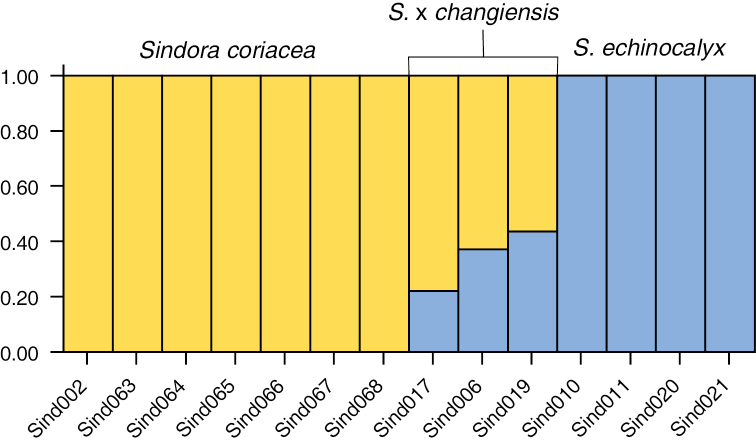
STRUCTURE plot generated for the optimal value of K = 2. Samples identified as *Sindoracoriacea* are wholly yellow, *Sindoraechinocalyx* samples are wholly blue, while the hybrid individuals of Sindora×changiensis have a mix of both yellow and blue.

The Neighbour-Net analysis in SPLITSTREE (Fig. 1) showed all three individuals of S.×changiensis to be located between *S.coriacea* and *S.echinocalyx* in the plot, which indicated that S.×changiensis was likely the result of hybridisation between the two parent species. The STRUCTURE analysis of the subsample of 10,000 SNPs also showed similar results at K = 2, where individuals of *S.coriacea* and *S.echinocalyx* were assigned to separate genetically distinct populations, and individuals of S.×changiensis were shown to be admixed between the two parents, as shown in Fig. 2, where the genetic composition of S.×changiensis is a mix of *S.coriacea* and *S.echinocalyx*. The optimal value of K for the STRUCTURE analysis, was also found to be 2, as this was the value with the highest ΔK value among the values for K = 2–5 that were calculated (Suppl. material 1: Fig. S1). The value of ΔK for K = 1 cannot be calculated, but K = 1 can be excluded as the optimal value of K because there are two known species in our sampling, hence the optimal value of K has to be at least 2. As such, both the STRUCTURE analysis and the ΔK calculation suggests that the number of populations that best explains the distribution of SNPs is 2, which corresponds to the two parent species *S.coriacea* and *S.echinocalyx*, and that S.×changiensis is the result of a hybridisation event between the two species. These results confirm the hybrid origin of S.×changiensis.

### ﻿Morphological analysis

**Figure 3.**
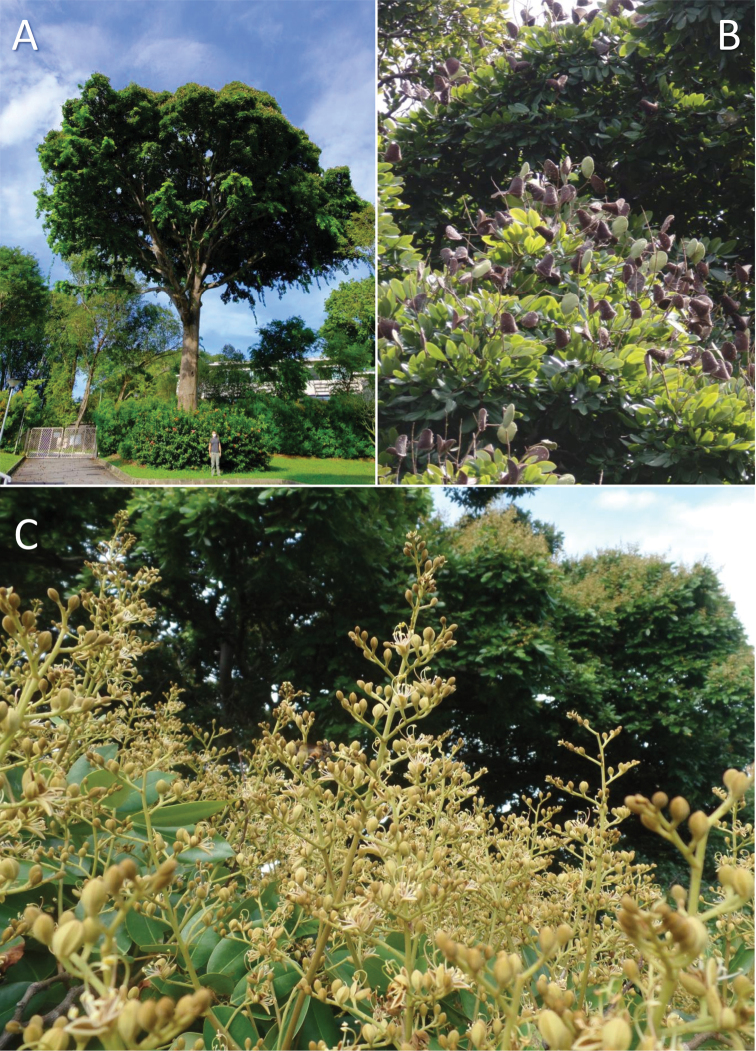
The hybrid tree Sindora×changiensis**A** overview of the 27 m tall tree **B** developing (green) and ripe (brown) pods on the tree **C** inflorescences of the tree. (Photos: **A** K.B.H. Er, **B, C** L.M. Choo).

**Figure 4.**
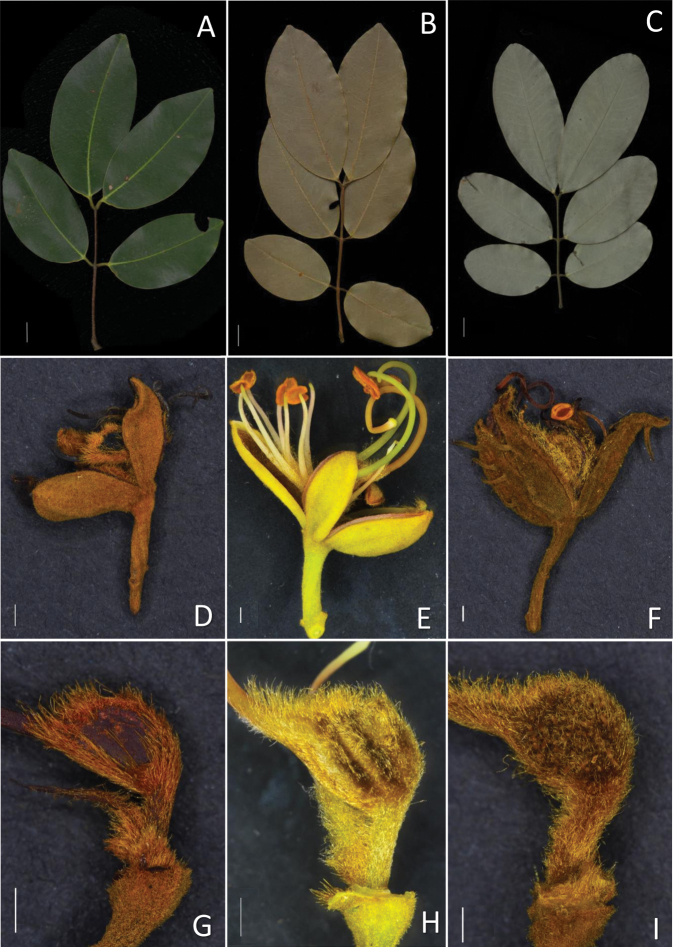
Comparisons of leaf and flower characters between Sindora×changiensis and its parent species. **A, B, C** leaves of (**A**) *S.coriacea*, (**B**) S.×changiensis and (**C**) *S.echinocalyx* respectively **D, E, F** flowers of (**D**) *S.coriacea*, (**E**) S.×changiensis and (**F**) *S.echinocalyx* respectively, showing the unarmed calyces of *S.coriacea, S.*×changiensis, and the spiny calyx of *S.echinocalyx***G, H, I** Ovaries of (**G**) *S.coriacea*, (**H**) S.×changiensis and (**I**) *S.echinocalyx* respectively, showing the glabrous patch in the centre for *S.coriacea*; the densely pubescent ovary for S.×changiensis except for the three stripes across the width; and the densely pubescent ovary for *S.echinocalyx*, with tiny protuberances visible on the surface, which will later on develop into the spines on the fruit pods. Scale bars: 1 cm (**A, B, C**); 1 mm (**D, E, F, G, H, I**). (Photos: L.M. Choo).

**Figure 5.**
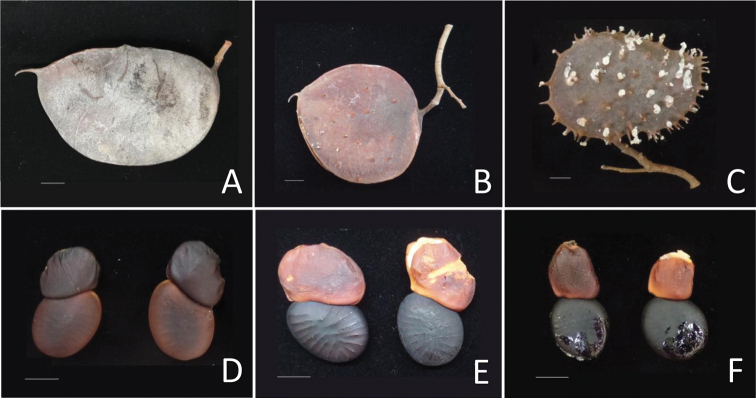
Comparisons of pod and seed characters between Sindora×changiensis and its parent species **A, B, C** pods of (**A**) *S.coriacea*, (**B**) S.×changiensis and (**C**) *S.echinocalyx* respectively, showing the unarmed pod of *S.coriacea*, the sparsely spiny pod of Sindora×changiensis, and the densely spiny pod of *S.echinocalyx***D, E, F** seeds of (**D**) *S.coriacea*, (**E**) S.×changiensis and (**F**) *S.echinocalyx* respectively. Scale bars: 1 cm (**A, B, C, D, E, F**). (Photos: L.M. Choo).

Morphological observations of the Sindora×changiensis along with *S.coriacea* and *S.echinocalyx* showed that S.×changiensis had characters, which were both intermediate between the two parents and also a mix of characters from either parent. The pods of S.×changiensis are sparsely spiny, which is intermediate between the non-spiny pods of *S.coriacea*, and the densely spiny pods of *S.echinocalyx*. The calyx of S.×changiensis is smooth and unarmed, which resembles *S.coriacea*. The ovary of S.×changiensis is densely villous all over and without spines on the surface, which is different from both *S.coriacea* and *S.echinocalyx*, where the ovaries are densely pubescent but with a glabrous patch in the centre, and densely villous all over but with small, blunt spines visible under the hairs, respectively. These differences are further detailed in Table 2 and depicted in Figs 3–5. A taxonomic treatment of the new taxon, and an updated key to the *Sindora* of Singapore and Peninsular Malaysia are also provided below.

**Table 2.** Differences between Sindora×changiensis and its parent species *S.coriacea* and *S.echinocalyx*.

Character	*Sindoracoriacea*	*Sindora×changiensis*	*Sindoraechinocalyx*
Stipules	Caducous, c. 4 mm long	Caducous, 1.2–1.4 cm long	Subpersistent, 0.8–1.8 cm long
Leaflet shape	Elliptic to ovate to broadly falcate, asymmetric with the midrib curved	Elliptic, slightly asymmetric with midrib slightly curved	Elliptic to obovate, slightly asymmetrical at the base, midrib generally straight
Leaflet apex	Bluntly acute to acuminate, rarely obtuse and sometimes slightly emarginate at the very tip	Acuminate to obtuse, with a very slight emarginate indent at the very tip	Apex rounded to obtuse and slightly emarginate at the very tip
Sepal size	4–5(–6.8) × 1.5–3 mm	8–9.3 × 3–5.2 mm	6–9 × 1.5–4.7 mm
Calyx surface	Unarmed	Unarmed	Armed all over with long, soft spines that are brittle when dry, spines up to 2 mm long.
Ovary surface	Densely pubescent around the edges and glabrous in the centre, surface unarmed.	Densely covered all over with long silky villous hairs, except in three strips on the surface where the hairs are less dense, spines not seen.	Densely villous, surface armed with small, blunt, spines that are visible under the hairs of the ovary
Ovary size	2.5–3.2 × 1.5–2 mm	3.5–4.5 ×2.8–3 mm	3.5–4 × 3–3.2 mm
Ovary stipe length	1.5–1.7 mm	1.8–2.2 mm	2.2–2.3 mm
Style length	9.5–10.5 mm	12–13.5 mm	9–12 mm
Pod surface	Unarmed or with few slightly raised warts	Sparsely armed with c. 20 or fewer slender spines which sometimes exude a clear resin	Densely armed with upright, regularly spaced spines, often with tipped with beads of dried resin at the ends of the spines
Aril	Aril semi-circular or trapezoid, chestnut-brown, 1.4–2 × 1.3–1.5 × 0.9–1 cm,	Aril trapezoid, yellowish brown to chestnut brown, 2.2–2.6 × 1.5–1.7 × 0.8–1 cm;	Aril narrowly trapezoid or rectangular, 1.3–1.5 × 1.3–1.8 × 0.6–0.8 cm,
Seed	1.9–2.5 × 1.4–1.8 × 0.8–0.1 cm, horizontal cracks on surface very faint and scarcely visible, chestnut brown in colour but becoming a darker shade of brown towards the centre of the seed.	2–2.5 × 1.7–1.9 × 0.8–0.9 cm, horizontal cracks on surface distinctly visible, uniformly black in colour.	1.9–2.2 × 1.1–1.9 × 0.6–0.9 cm, horizontal cracks on surface very faint and scarcely visible, uniformly black in colour.

### ﻿Taxonomic treatment

#### ﻿ Sindora × changiensis

Taxon classificationPlantaeFabalesFabaceae

L.M.Choo, Loo, W.F.Ang & K.Er nothosp. nov.

B00B217B-24D9-5AAE-8BC3-32F3797DC8B4

Figs 3 , 4 , 5

= Sindoracoriacea (Baker) Prain × Sindoraechinocalyx Prain.

##### Diagnosis.

Pod intermediate in character between the two parents, with a smooth surface like that of *S.coriacea* coupled with sparsely-set and fine spines which are much less dense than in *S.echinocalyx*. Flower calyx entirely smooth and without prickles, resembling *S.coriacea*. Ovary lacking the hairless patch in the centre, which is the case for *S.coriacea*. Instead, it is entirely pubescent with fine adpressed hairs, but without the minute protuberances or prickles that are seen in *S.echinocalyx*.

##### Type.

Singapore: Changi: 503 Cranwell Road, 1°23.335'N, 103°58.618'E, 6 May 2021, Choo et al., SING2021-265 (holotype SING, isotypes (BKF, K, KEP, L)).

Tree up to 27 m tall, dbh up to 1.5 m, bole columnar, with slightly raised rings around the girth, not buttressed, bark grey to blackish, slightly cracked or flaky. Stipules early caducous, only present in young parts, semicircular, 1.2–1.4 cm long. Leaves compound, paripinnate, 3–4 jugate, rachis puberulous, 3.5–5.6 cm long; petiole 2–3 cm long; petiolules 4–5 mm long, puberulous, grooved, greenish brown when fresh but drying dark brown to black. Leaflets opposite, coriaceous, elliptic, slightly asymmetric with midrib slightly curved, 3.5–6.8 × 2.3–3.5 cm, increasing in size up the rachis, base rounded to obtuse, apex acuminate to obtuse, with a very slight emarginate indent at the very tip; upper surface slightly glossy when fresh, but reticulations become conspicuous when dry, entirely glabrous, midrib flat to slightly sunken; lower surface glaucous, puberulous with tiny short golden hairs, midrib raised and also puberulous; thickened marginal vein either glabrous or minutely puberulous; reticulations clear and raised on both the upper and lower surfaces; one gland present on the tip of the midrib on the lower surface, another present on the thickened marginal vein close to the base of the leaflet. Inflorescence paniculate, both terminal and axillary, but mostly concentrated on the crown, growing from old stems where inflorescence branches from the previous year have been shed, flowering rachises long and stout, measuring 11.5–25 × 5–8 cm, side branches straight but bearing scars where the flowers are attached, branches flexible but held erect in fresh specimens. Both flowering rachis and branches completely pubescent with short golden adpressed to upright hairs. Bracts not seen, caducous; bracteoles ovate, c. 2.5 × 1.3 mm, pubescent on both surfaces, caducous, only seen in inflorescences where the buds are still small and developing. Pedicels 4.5–6 mm long, pubescent, receptacle short, 1–1.5 mm long; buds obovoid to ellipsoid, suture lines of the sepals becoming evident as the bud matures, measuring 6–7.5 × 4.5–5.5 mm when mature just before anthesis. Flowers strongly zygomorphic. Sepals 4, unequal, lanceolate to elliptic, 8.0–9.3 × 3.0–5.2 mm, outer surface pubescent with small golden hairs, unarmed, inner surface densely covered with long golden brown tightly adpressed hairs. Petal 1, not exserted but nestled within the largest sepal during anthesis, rolled up and containing a drop of sweet floral-scented nectar, c. 7.5 × 2.2 mm when rolled up, top of petal with a well-defined hood fringed with long villous hairs which narrows off with the lower half of the petal with inrolled sides forming a closed tube; outer surface glabrous at the top and down the middle, densely pubescent at the sides and the lower half of the petal; inner surface glabrous; margins villous, colour pink tinged with green at the tip. Stamens 10, diadelphous, united basal portion of the stamens 2.5–3 mm long; two largest filaments 12–15 mm long, the seven in the middle of the bundle 6–7 mm long; two largest anthers elliptic, 2.5–2.7 × 1.7–1.9 mm, the others smaller and heart shaped, 1.6–2 × 1–1.4 mm, all nine mentioned here with visible pollen; final stamen on the other side of the flower is a staminode, 6–7.5 mm long but without a fertile anther. Ovary rhomboidal, densely covered all over with long silky villous hairs, except in three strips on the surface where the hairs are less dense; 3.5–4.5 × 2.8–3 mm, stipe 1.8–2.2 mm long, style glabrous except for the base where it has villous hairs like the rest of the ovary, 12–13.5 mm long, pale yellow green tinged with pink at the base, stigma capitate with small sticky papillate protuberances, c. 0.6 mm diameter. Pod a flattened, elliptic, rhomboidal or ovate two-valved dehiscent pod, surface sparsely armed with c. 20 or fewer slender spines that sometimes exude a clear resin; surface beneath the spines smooth and puberulous with short golden hairs, 7–8 × 6–6.5 cm, stipe 8–9 mm, beak 9–10 mm. Seed 1, aril trapezoid, yellowish brown to chestnut brown, 2.2–2.6 × 1.5–1.7 × 0.8–11 cm; seed 2–2.5 × 1.7–1.9 × 0.8–0.9 cm, surface smooth with fine horizontal cracks, black in colour.

##### Distribution.

The hybrid is likely endemic to Singapore. It is only known to occur naturally in Changi, which is at the north-east coast of Singapore, although the offspring of the tree has been propagated and planted elsewhere in Singapore as roadside trees.

##### Etymology.

Latin, -*ensis* = from, meaning “from Changi”.

##### Habitat and ecology.

The species is part of the remnant vegetation of tropical lowland forest that was once present in the area, before it was cleared.

##### Phenology.

Flowers from April to May, and fruits in August.

##### Conservation.

Only a single tree of S.×changiensis is known to occur from the wild in Singapore, although the offspring of this tree have been planted elsewhere in Singapore as roadside trees.

##### Taxonomic notes.

In *Sindora*, the leaves of seedlings, saplings and water shoots of mature trees often have a morphology different from that of the mature leaves from the crown of the tree. The leaves of *Sindora* seedlings, saplings and water shoots are usually larger in size and are pubescent on the underside and along the leaflet margins, and the shape of the leaf and the leaf apex may differ somewhat from the mature leaves (de Wit 1949; Choo and Ngo 2020). For the identification of this hybrid, pod characters are the most diagnostic, followed by flower characters, although fallen mature leaflets picked from below the tree may be useful in supplementing the fruit and flower characters.

### ﻿Updated key to the *Sindora* species in Singapore and Peninsular Malaysia

**Table:** 

1	Leaflets broadly elliptic, apex strongly emarginate, midrib on the lower surface with a gland located 1–3 mm away from the tip of the leaflet; calyx warty with small spines at the apex	***S.siamensis***
–	Leaflets falcate, elliptic, obovate or lanceolate, apex if emarginate only very slightly notched at the tip, midrib on the lower surface with a gland at the very tip of the leaflet; calyx never warty, either unarmed or armed with spines	**2**
2	Lower surface of leaflets densely pubescent or tomentose, distinctly velvety or rough to touch; leaves 4–6-jugate; rachis of young leaflets, inflorescence and stipe of pods densely tomentose with reddish brown hairs	***S.velutina***
–	Lower surface of leaflets puberulous to glabrescent to glabrous, may be slightly rough like fine sandpaper but never velvety; leaves (2–)3–4-jugate; rachis of young leaflets puberulous to glabrous, inflorescence or stipe of pods pubescent to tomentose with golden brown hairs	**3**
3	Lower surface of leaflets glabrous or only sparsely puberulous at the base in mature leaflets except for the midrib which is usually puberulous; pods unarmed	***S.coriacea***
–	Lower surface of leaflets entirely puberulous with small, thin adpressed or strigose hairs in mature leaflets; pods armed or sparsely armed	**4**
4	Calyx entirely smooth and without spines; pods sparsely armed, usually with c. 20 spines or much less on each surface	**S.×changiensis**
–	Calyx armed, either only on the upper half or on the entire surface; pods armed with more than 20 spines on each surface	**5**
5	Leaflets without raised reticulations above, upper surface smooth and glossy and shining; calyx armed only on the upper half or on the very tip of the bud, with small spines less than 1 mm long	***S.wallichii***
–	Leaflets with raised reticulations above, upper surface not glossy; calyx armed all over the exterior of the bud with long soft spines which are brittle when dry, spines up to 2 mm	***S.echinocalyx***

## ﻿Discussion

**Figure 6.**
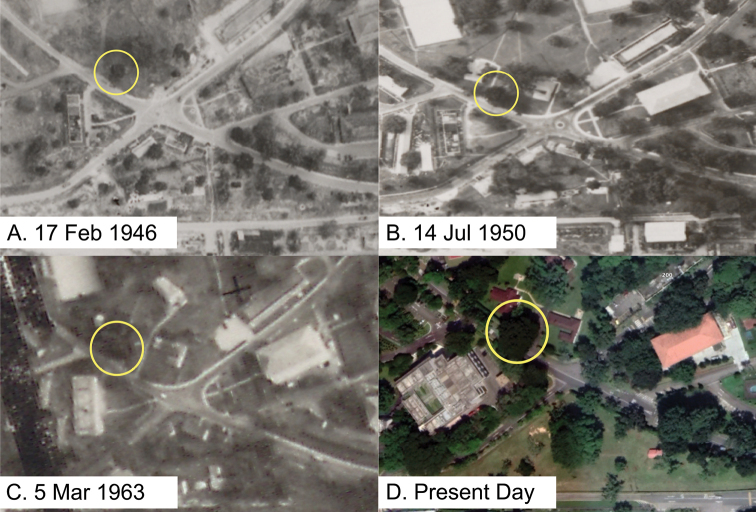
Aerial photo of the Changi area from various years showing Sindora×changiensis circled in yellow **A** aerial photo on 17 Feb 1946, which is the earliest archival aerial photo of the Changi area, showing the tree as part of the rainforest remnant **B** aerial photo on 14 Jul 1950, showing the erection of the Cranwell bungalows to the left and right of the tree **C** aerial photo on 5 Mar 1963 **D** present day aerial photo showing the tree. (Images: **A–C** aerial photographs by the British Royal Air Force between 1940 to 1970s, from a collection held by the National Archives of Singapore. Crown copyright, reproduced in part **D** imagery 2021 Maxar Technologies, Map data 2021 Google).

Sindora×changiensis is the first recorded instance of natural hybridisation in the genus *Sindora*, and is currently known to be endemic to Singapore. Both of the parent species, *S.coriacea* and *S.echinocalyx*, are native to Singapore. *S.echinocalyx* is known to occur in coastal hills and heath forests in Peninsular Malaysia (Saw 2010) and had been recorded in Changi in the past based on an herbarium specimen (*Bakar s.n.*, Year 1893, Changi, SING[SING0044593]). In addition, *S.coriacea* is a native of lowland rainforests in Singapore (Chong et al. 2009; Choo and Ngo 2020). Located at the north-eastern coast of Singapore, Changi was known to be dominated by lowland rainforest and was previously designated as a forest reserve in 1884, before it was largely cleared by the British to make way for military barracks and an airbase from 1927 to 1941 (Probert 1965; Corlett 1992). However, there are presently still a few large trees of lowland rainforest species in Changi, which are remnants of the original rainforest vegetation, such as *Shoreagibbosa*, *Dipterocarpussublamellatus* and *Glutamalayana*. Based on early aerial photographs of the site in 1946 and 1950, it showed that the mature *Sindora×changiensis* tree was part of a remnant rainforest patch that was retained as part of the grounds of the existing bungalow (503 Cranwell Road) that served as the residence of senior British military officers based at Changi (National Archives of Singapore records) (Fig. 6). Carbon dating also estimated that the tree is at least approximately 226 to 364 years old, further suggesting that the tree was naturally occurring in-situ and could not have been planted (National Parks Board, unpublished data).

This hybrid individual was observed to have been pollinated by the giant honey bee (*Apisdorsata*). These bees are known to be able to transfer pollen over sizeable distances, and have also been inferred to facilitate pollen dispersal of another leguminous tree species, *Koompassiamalaccensis*, between rainforest patches across a distance of more than 2.5 km within the urban landscape of Singapore (Noreen and Webb 2013; Noreen et al. 2016). Given that these bees are likely to be pollinators of other *Sindora* species, it is therefore possible that this hybrid could have come about by the transfer of pollen between *S.coriacea* and *S.echinocalyx* trees at Changi in the past.

In both the Neighbour-Net (Fig. 1) and STRUCTURE analyses (Fig. 2), the proportion of *S.coriacea* in the genetic composition of the mature hybrid tree (Sind019) is higher than that of *S.echinocalyx*. This might suggest that *S.coriacea* is the maternal parent, which contributes both plastid and nuclear genetic material to the hybrid (Greiner et al. 2015). Another possibility is that the hybrid tree or its seedlings (Sind006, Sind 017) could be backcrossed to *S.coriacea*, which could explain the larger genetic contribution of *S.coriacea* to the hybrid. However, further studies including the sequencing of the chloroplast genome and the sampling of more hybrid offspring will be necessary to ascertain this.

This hybrid is fertile, with extensive flowering and fruit set happening once a year (Fig. 3), based on field observations from August 2020 to September 2021. The hybrid characters are also stable, as an offspring of the hybrid tree which was planted in 2001 has also been observed to produce fruits with a similarly sparse distribution of spines (Choo SING2021-677, 17 Sep 2021, outside Changi Chapel & Museum, SING). While this is unusual as hybrids often have lower fitness and are usually sterile (Ashton 1969; Hopkins 2013; reviewed in Schley et al. 2020), natural *Shorea* hybrids in the rainforest of Singapore have been documented to be fecund (Kamiya et al. 2011). The growth rates and survivability of *S.curtisii* × *S.leprosula* seedlings were also found to be comparable to the parent species, albeit with slightly slower growth rate than *S.leprosula*, suggesting similar fitness to that of parent species in the rainforest environment (Kenzo et al. 2016, 2019). There have also been suggestions that environmental disturbance could potentially result in less favourable conditions for parental species and give rise to conditions that favour natural hybrids, with implications for conservation (Levin et al. 1996; Rieseberg and Carney 1998). This could have enabled Sindora×changiensis to establish and persist over time, even as its habitat changed drastically from a primary rainforest to an urban environment. More research could be done to further examine the mechanisms giving rise to this fertile hybrid, including the possibility of backcrossing with its parent species, as well as the effects of this on the evolutionary trajectory and the genetic diversity of *Sindora* in rainforest patches within anthropogenic landscapes.

## Supplementary Material

XML Treatment for Sindora × changiensis

## Supplementary materials

Supplementary material 1 Figure S1 Data type Image. Explanation note ΔK value for the values of K = 2–5 for the 30 iterations of STRUCTURE conducted. The value of ΔK for K = 1 cannot be calculated. The highest value of ΔK at K = 2 shows that 2 is the optimal value of K for the STRUCTURE analysis. https://binary.pensoft.net/file/649934This dataset is made available under the Open Database License (http://opendatacommons.org/licenses/odbl/1.0/). The Open Database License (ODbL) is a license agreement intended to allow users to freely share, modify, and use this Dataset while maintaining this same freedom for others, provided that the original source and author(s) are credited.Le Min Choo, Adrian Hock Beng Loo, Wee Foong Ang, Kenneth Boon Hwee Er
