# Supplementary material for: ﻿A natural hybrid of Sindora (Fabaceae, Detarioideae) from Singapore
Source: PhytoKeys. 2022 Feb 23;190:87–102. doi: 10.3897/phytokeys.190.79185 (PMC8891240; doi:10.3897/phytokeys.190.79185)
Supplement: Supplementary material 1 — Figure S1 [file phytokeys-190-087-s001.docx]

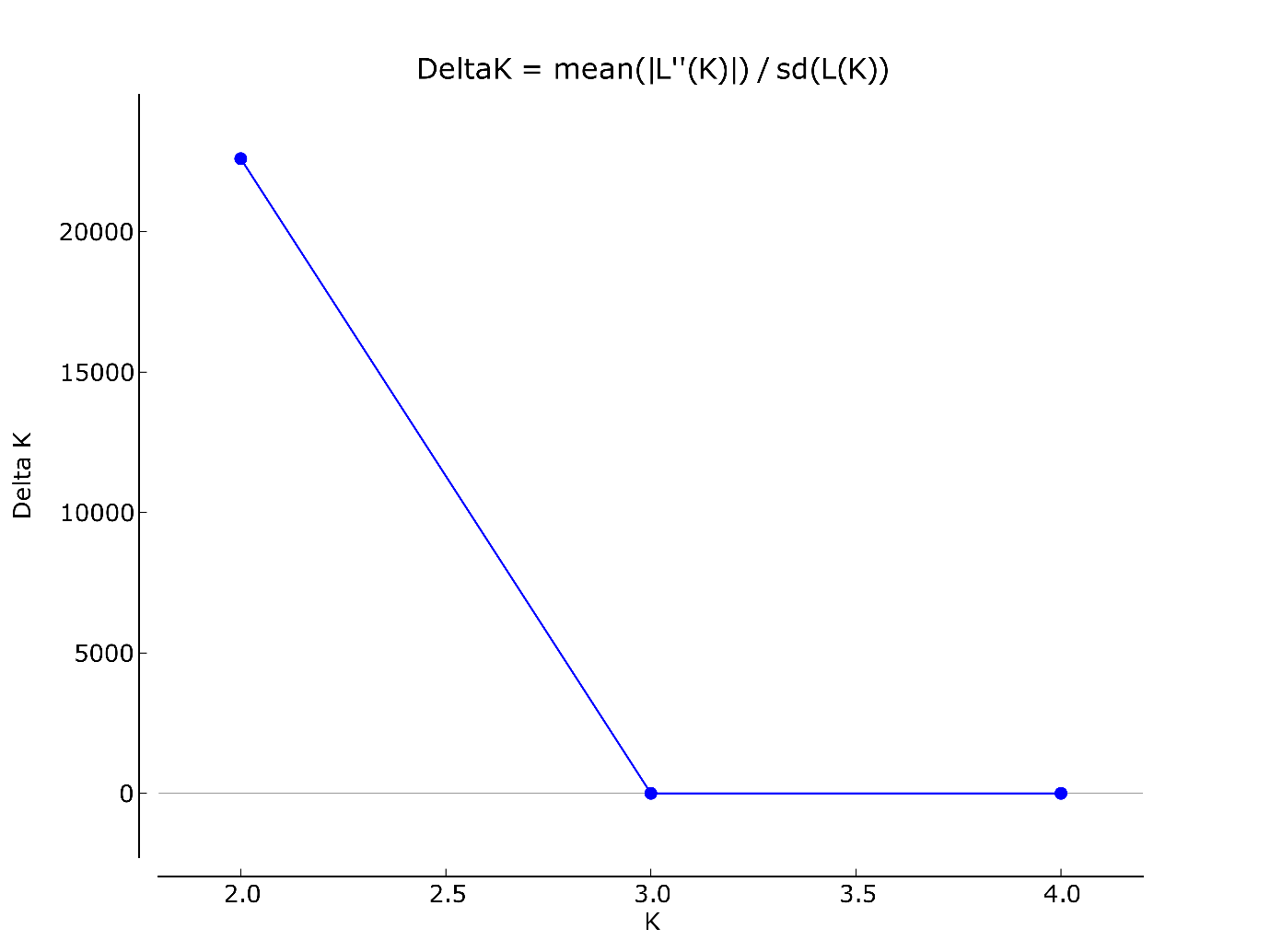
Supplementary Fig. 1: ΔK value for the values of K=2–5 for the 30 iterations of STRUCTURE conducted. The value of ΔK for K=1 cannot be calculated. The highest value of ΔK at K=2 shows that 2 is the optimal value of K for the STRUCTURE analysis.
